# Understanding and Predicting Population Response to Anthropogenic Disturbance: Current Approaches and Novel Opportunities

**DOI:** 10.1111/ele.70198

**Published:** 2025-08-22

**Authors:** Cassie N. Speakman, Sarah Bull, Sarah Cubaynes, Katrina J. Davis, Sébastien Devillard, John M. Fryxell, Cara A. Gallagher, Elizabeth A. McHuron, Kévan Rastello, Isabel M. Smallegange, Roberto Salguero‐Gómez, Elsa Bonnaud, Christophe Duchamp, Patrick Giraudoux, Simon Lacombe, Courtney J. Marneweck, Louis Schroll, Adrien Tableau, Sandrine Ruette, Olivier Gimenez

**Affiliations:** ^1^ FRB‐CESAB Montpellier France; ^2^ CEFE, Univ Montpellier, CNRS, EPHE, IRD Montpellier France; ^3^ Department of Biology University of Oxford Oxford UK; ^4^ Universite Claude Bernard Lyon 1, LBBE, UMR 5558, CNRS, VAS Villeurbanne France; ^5^ Department of Integrative Biology University of Guelph Guelph Ontario Canada; ^6^ Department of Biology University of Victoria Victoria British Columbia Canada; ^7^ Department of Ecoscience Aarhus University Aarhus Denmark; ^8^ Cooperative Institute for Climate, Ocean, and Ecosystem Studies University of Washington Seattle Washington USA; ^9^ School of Natural and Environmental Sciences Newcastle University Newcastle upon Tyne UK; ^10^ Laboratory Ecology Systematic and Evolution, UMR 8079 Université de Paris‐Saclay Gif‐sur‐Yvette France; ^11^ French Biodiversity Agency (OFB) France; ^12^ Chrono‐Environment Lab University Marie and Louis Pasteur/CNRS Besançon France; ^13^ Giraffe Conservation Foundation Windhoek Namibia; ^14^ Applied Behavioural Ecology ER Unit University of South Africa Johannesburg South Africa

## Abstract

Effective conservation of biodiversity depends on the successful management of wildlife populations and their habitats. Successful management, in turn, depends on our ability to understand and accurately forecast how populations and communities respond to human‐induced changes in their environments. However, quantifying how these stressors impact population dynamics remains challenging. Another significant hurdle at this interface is determining which quantitative approach(es) are most appropriate given data types, constraints and the intended purpose. Here, we provide a cross‐taxa overview of key methodological approaches (e.g., matrix population models) and model elements (e.g., energetics) that are currently used to model the effects of anthropogenic disturbance on wildlife populations. Specifically, we discuss how these modelling approaches differ in their key assumptions, in their structure and complexity, in the questions they are best poised to address and in their data requirements. Our intention is to help overcome some of the methodological biases that might persist across taxonomic specialisations, identify new opportunities to address existing modelling challenges and improve scientific understanding of the direct and indirect impacts of anthropogenic disturbance. We guide users through the identification of appropriate model configurations for different management purposes, while also suggesting key priorities for model development and integration.

## Graphical abstract

Here, we provide a cross‐taxa overview of key methodological approaches and model elements that are used to model the effects of human disturbance on wildlife populations. We discuss how these modelling approaches differ in their key assumptions, their structure and complexity, the questions they are best poised to address and their data requirements. We guide users through the identification of appropriate model configurations for different management purposes, while also suggesting key priorities for model development and integration.

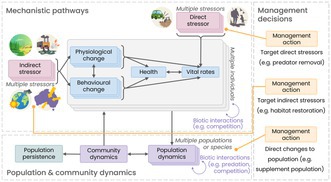

## 1 Introduction

As the extent and magnitude of human activity continues to expand (IPBES 2019), the urgency to understand how wildlife populations respond to anthropogenic change is accelerating (Larson et al. 2016; Venter et al. 2016). This information is crucial for effective management and conservation policies (Pimm et al. 2014). Ecologists have long tried to understand and predict the impacts of human disturbance on wildlife populations and communities through the use of quantitative modelling approaches (Beissinger and Westphal 1998; Getz and Haight 1989). However, stressors rarely occur in isolation, with animal populations often exposed to multiple direct (e.g., harvesting; Kays et al. 2017) and indirect stressors (e.g., habitat fragmentation, Smith et al. 2019). Although it is relatively straightforward to determine how individual stressors impact populations, the complex ways that multiple stressors interact make it challenging to predict their combined effect on a population (Paniw et al. 2021). As a result, there has been an increasing focus on understanding the population‐level effects, such as changes in population dynamics, geographical distribution and/or population persistence, that can result from exposure to multiple stressors (e.g., Gosselin et al. 2015; Daversa et al. 2025; Pirotta et al. 2019; Galic et al. 2018).

**FIGURE 1:**
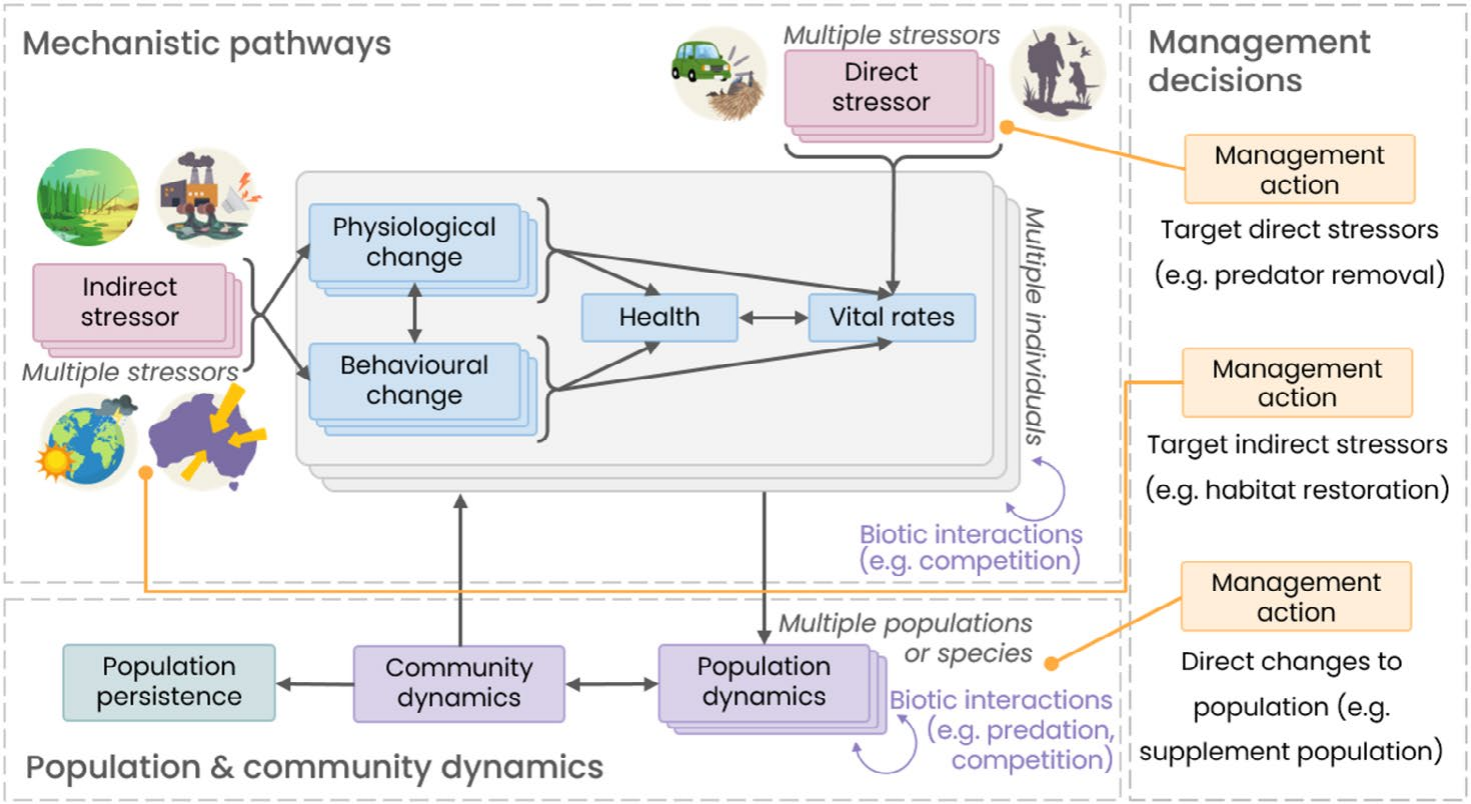
Conceptual framework for assessing the individual‐, population‐ and community impacts of human disturbance and management actions on animal populations. Adapted and extended from (National Academies of Sciences, Engineering, and Medicine 2017).

Oftentimes, disturbances have indirect effects on populations, such as through changes in food intake, which may lead to changes in energy balance and/or body condition (Parker et al. 2009) or through exposure to pathogens or pollutants, which may impact immune status (Charbonnel et al. 2008). These indirect effects can compound, leading to impacts on vital rates (e.g., survival, reproduction) that then shape population dynamics (Figure 1, Box 1). Several conceptual frameworks have been developed that explicitly consider these indirect effects (Johnston et al. 2019; National Academies of Sciences, Engineering, and Medicine 2017; Simmons et al. 2021; Urban et al. 2022). Johnston et al. (2019) and National Academies of Sciences, Engineering, and Medicine (2017) describe the individual‐ and population‐level impacts of disturbance, while Urban et al. (2022) and Simmons et al. (2021) also consider community‐ and ecosystem‐level consequences of disturbance, and prioritisation of management actions (only Urban et al. 2022). However, being solely conceptual frameworks, they lack information on the specific approaches that can be used to model population‐level impacts of disturbance.

BOX 1 **From individuals to communities–pathways for human impacts on wildlife.**

The impacts of human disturbance and many other stressors occur not only via directly altering the vital rates (e.g., survival, fecundity) of a population but also indirectly via changes in the behaviour and/or physiology of individuals (National Academies of Sciences, Engineering, and Medicine 2017). When sustained, stressors can lead to chronic impacts that erode the health and immune status of individuals. Stressors may also have an acute impact on vital rates, such as mortality resulting from an injury. These individual level responses can then be scaled up to the population‐level by considering multiple individuals and ideally their interactions within a population. Representing multiple interacting stressors requires the explicit consideration of multiple mechanistic pathways through which these stressors are acting. For example, the European mink (see 3.2) is exposed to a range of stressors including the direct effects of road mortality, which relates to individual movement patterns and habitat connectivity, and indirect effects of wetland loss and competition with American mink, resulting in behavioural and energetic impacts on the population.

Community and ecosystem dynamics are important when identifying appropriate conservation or management strategies of specific populations, since a lack of consideration can lead to adverse management outcomes (e.g., Buckley and Han 2014; Zavaleta et al. 2001). Despite this, these dynamics are rarely accounted for in disturbance models, particularly mechanistic models, likely due to the increasing complexity when considering multiple populations or species. Nonetheless, the increasing impact of human activities on ecological systems and their continued degradation (IPBES 2022) emphasises the need for human disturbance modelling to incorporate community dynamics. There are many approaches that can be used to account for community dynamics (Geary et al. 2020), which can be integrated with other quantitative approaches to leverage off the benefits of each approach. For example, individual‐based models can be combined to create community models (e.g., Radchuk et al. 2013) or can be integrated within matrix community models (Lytle and Tonkin 2023), depending on the specific data available. Community models also lend themselves well to energetics approaches (Szangolies et al. 2024), permitting mechanistic evaluation of community dynamics.

The mechanistic pathways of impact are not only relevant for studying the impacts of stressors on wildlife; they can also be used to measure the effectiveness and appropriateness of management actions. By considering how management actions are intended to impact the target population, such as through improved resource availability, ecological models can help identify if management strategies will have their intended outcome or if alternative strategies may be more worthwhile. This may be particularly beneficial when considering community dynamics, where unintended consequences are more likely to occur.

Given the complexity of ecological systems, computational modelling approaches play an important role in understanding and managing ecosystems by clarifying the key mechanisms that might explain the behaviour of ecological systems (Schmolke et al. 2010). The main advantage of computational modelling approaches to management decision making is their ability to predict the magnitude of effect of alternative scenarios based on underlying processes, which is rarely possible through empirical studies alone (Skogen et al. 2024). Many approaches are available for modelling the impacts of human activity on animal populations—including individual‐based, demographic, range dynamics and community models—each with their own assumptions and caveats. The approaches differ in which processes are represented (and how), their spatio‐temporal scales, data requirements and in which questions they are best poised to answer. Inappropriate choices in modelling approach or structure could compromise the ability to make reliable predictions (Sirén et al. 2025), creating greater uncertainty when deciding on management strategies.

Despite the vast array of modelling approaches available, several key challenges remain when trying to predict the population‐level impacts of human activity on wildlife: (1) combining disparate data streams collected at different spatio‐temporal scales; (2) providing scientifically informed management advice for populations when limited empirical data are available, as is often the case; (3) managing uncertainty when uncertainty is everywhere; and (4) deciding which approach(es) to use given the available data and the question at hand. These challenges are exacerbated when considering community dynamics, which is often necessary to accurately predict the implications of human disturbance and potential management strategies on ecological communities (Buckley and Han 2014; Zavaleta et al. 2001). While challenges 1–3 have received considerable attention (e.g., Nichols 2021; Simmonds et al. 2024; Zipkin et al. 2019; Fletcher Jr. et al. 2019), guidance on modelling choices for assessing and predicting population‐level impacts of human disturbance has received less attention (but see Accolla et al. 2021; Hunter‐Ayad et al. 2020; Thompson et al. 2021; Briscoe et al. 2019).

**FIGURE 2:**
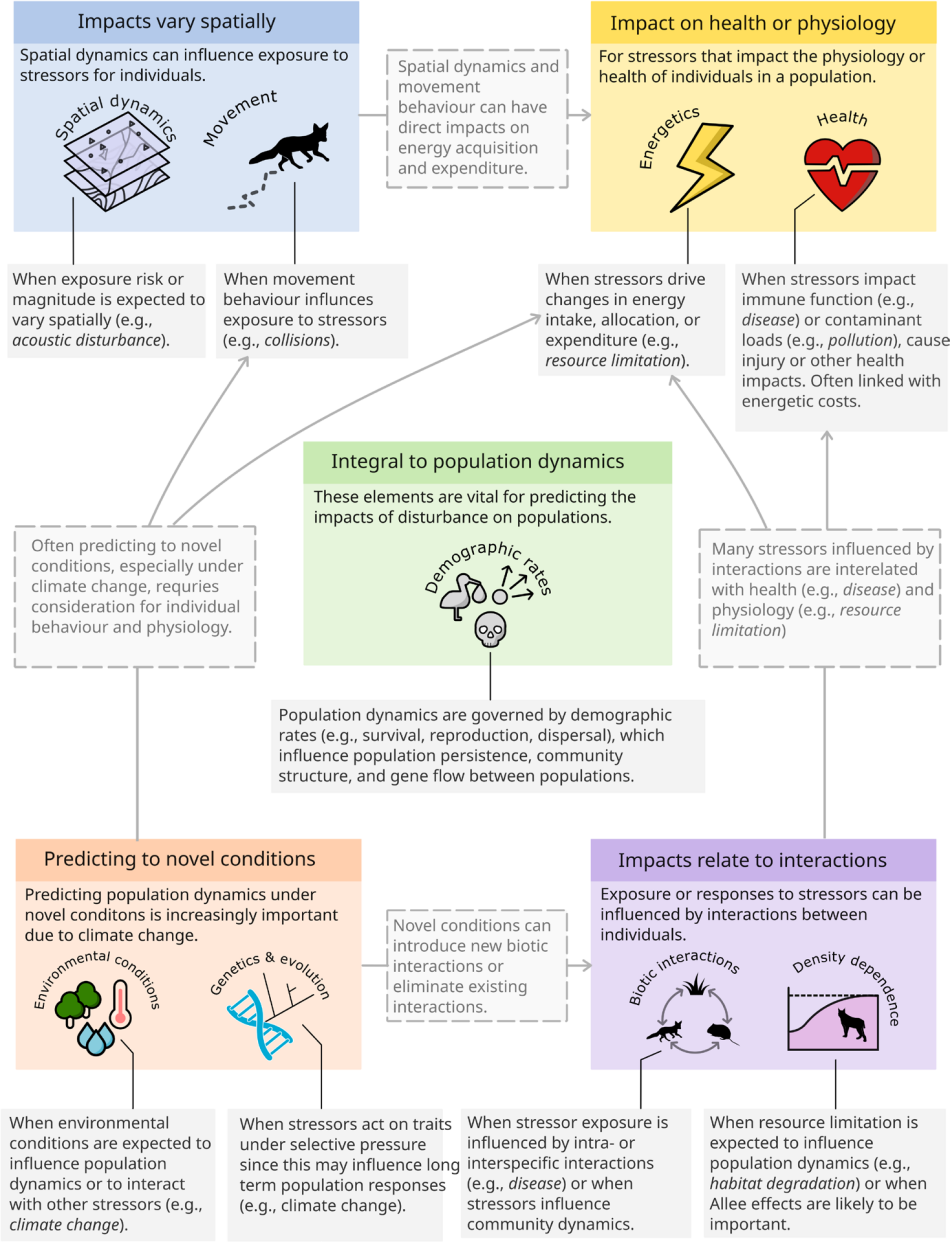
Considerations for ecological processes or mechanisms that should be considered depending on how disturbances impact a population. These considerations are non‐sequential and often interrelated, as indicated by the dashed boxes. Coloured boxes describe the different ways a population could be impacted or indicate key requirements. Grey boxes provide brief descriptions of when an ecological process may be relevant to include in a model.

**FIGURE 3:**
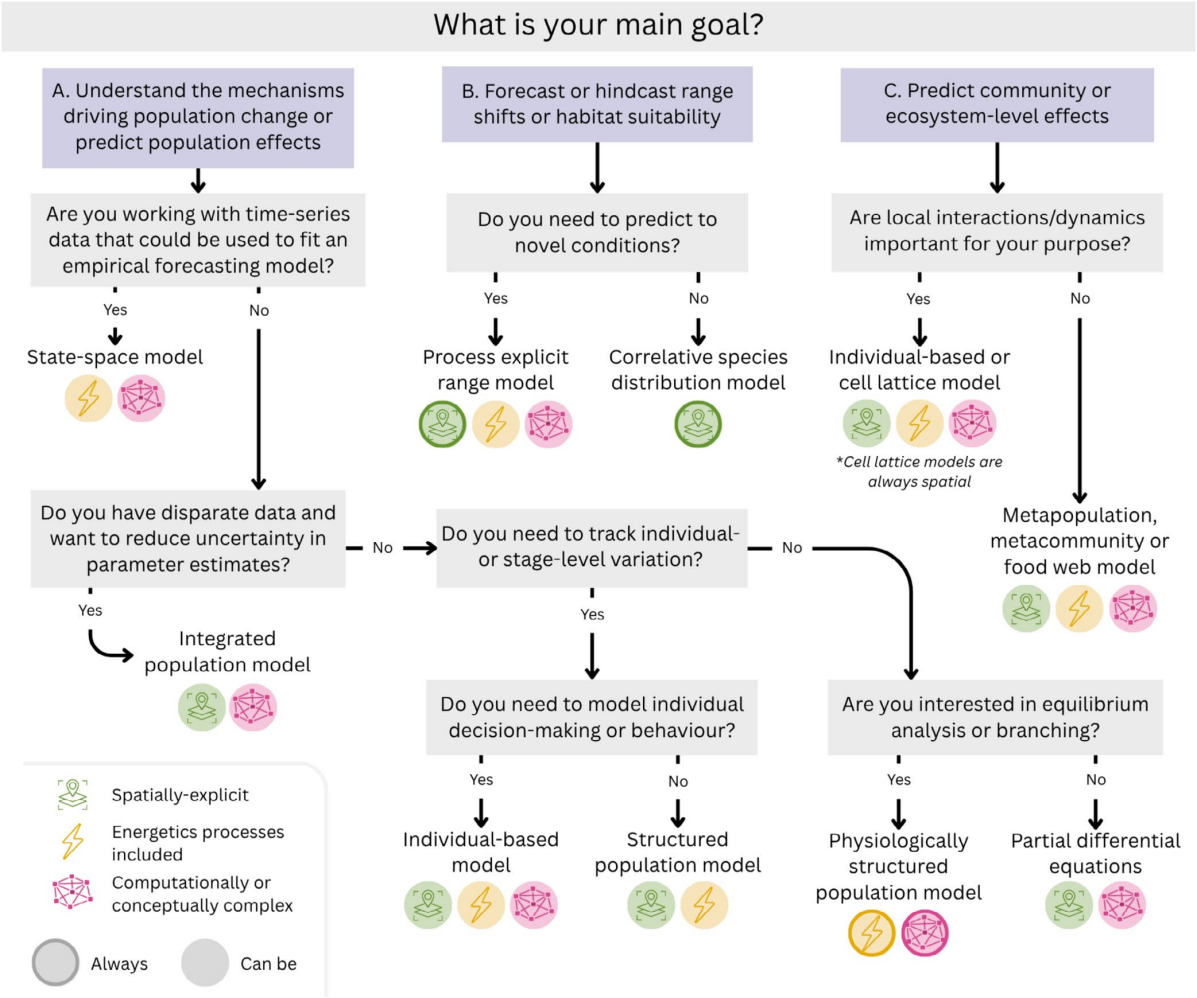
Decision tree for identifying appropriate modelling approaches depending on the primary research goal. Note, these approaches are limited to the modelling approaches discussed within this paper and the recommendations are under the assumption that data availability is not a primary limiting factor. Structured population models include matrix population models and integral projection models. For each approach, we indicate whether the approach is spatially‐explicit (green icon) or complex (pink icon), whether energetics processes can be incorporated (yellow icon) and whether is it always the case (solid outline on icon) or can be (no outline).

**FIGURE 4:**
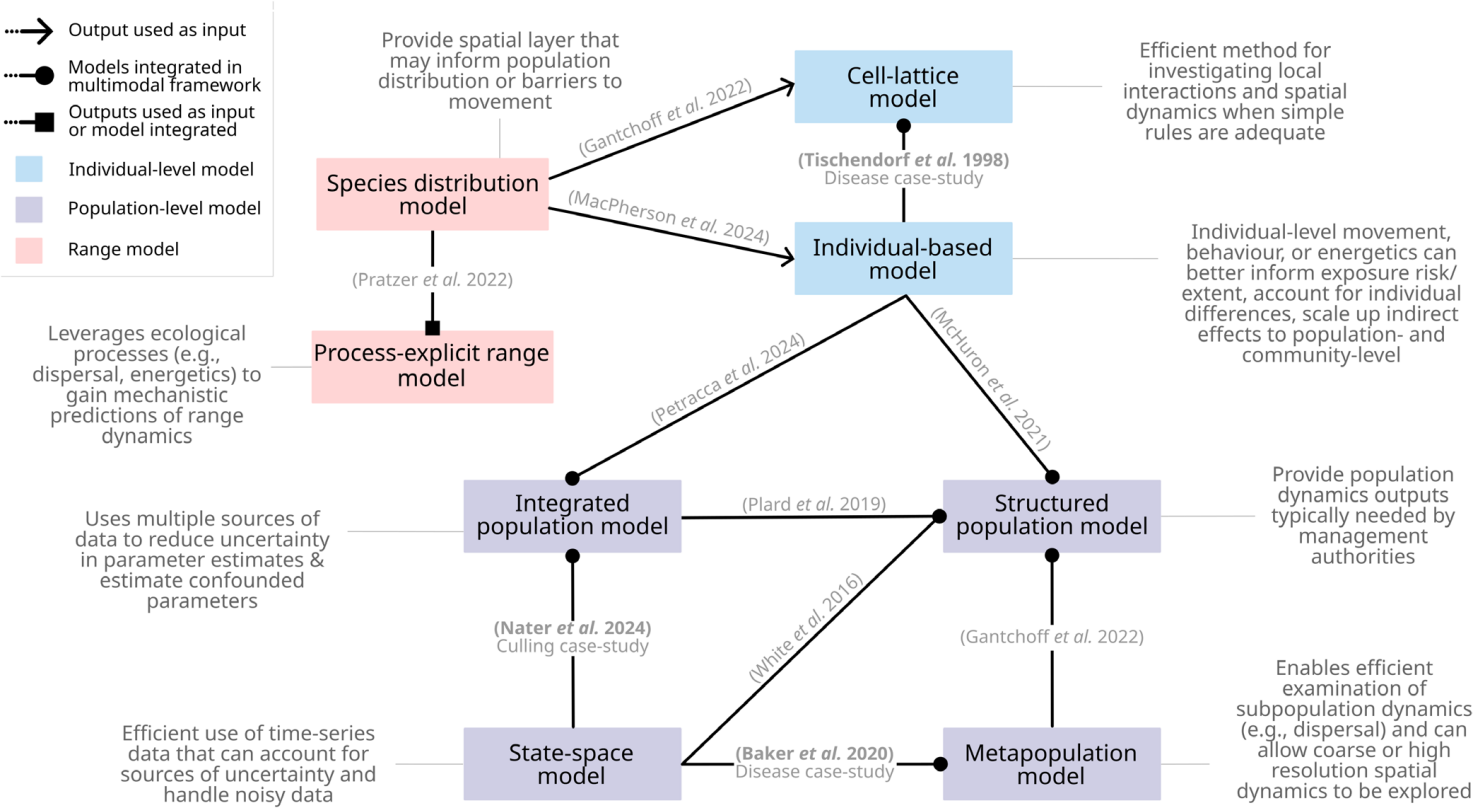
Overview of common or promising modelling integrations for disturbance ecology. Modelling approaches are coloured according to the ecological scale that disturbance impacts are usually modelled (i.e., geographical range–pink; individual‐level–blue; population‐level–purple). Descriptions of why each approach may be beneficial to integrate are attached to each modelling approach. Examples of integrations are indicated along the connecting arrows, with the type of integration indicated by the arrow head (arrow–the output of one model is used as an input for the other model; closed circle–both models are integrated within a single multimodal framework with feedback between components; closed square–integration type can be as for the arrow or closed circle depending on the specific modelling approach or research question).

Here, we present an overview of key approaches available to model human impacts on animal populations. In doing so, we aim to provide resources for new studies to identify suitable methods and help overcome taxonomic or research domain biases in model development. As part of this effort, we highlight relevant ecological processes to consider including (Figure 2), provide a decision tree to identify suitable modelling approaches (Figure 3), identify some common model integrations (Figure 4) and summarise the data requirements and anticipated outputs of each modelling approach (Table S1). We use case‐studies on red fox ( *Vulpes vulpes* ) and European mink ( *Mustela lutreola* ) to illustrate some of the considerations made during model development and the strategies used to overcome the limitations of different modelling approaches, including model integration and energetic modelling. Further, we extend existing conceptual frameworks for understanding the impacts of human disturbance by incorporating community‐level responses to multiple stressors as well as the pathways by which management actions can influence populations (Figure 1, Box 1). The information presented here can be used to identify appropriate model configurations for different research and management purposes, while also suggesting key priorities for future model development and integration.

### 1.1 Representing the Mechanistic Pathways of Human Impacts

Predicting the impacts of indirect or multiple stressors on animal populations necessitates explicit model representation of the biological processes, or mechanisms, through which stressors act. Most stressors have impacts at multiple ecological scales (i.e., individual, population, community) but the mechanisms driving those impacts may be isolated to a single ecological scale (e.g., physiological effects on individuals) (Simmons et al. 2021). By focusing on the scale at which stressors act, we can better identify which processes are most important for predicting disturbance impacts. Some processes are already well represented in many of the models used in disturbance ecology (e.g., demography) due to their vital role in population dynamics. There is little guidance, however, on which processes are important to consider when developing predictive models for animal populations exposed to disturbance (but see Urban et al. 2016).

In Figure 2, we highlight the key processes relevant to understanding the impacts of disturbances, emphasising the specific contexts in which each process becomes particularly important. Demographic processes are fundamental for predicting disturbance effects, as they directly influence population dynamics, gene flow and community structure. For stressors with spatially variable impacts—such as vehicle collisions or acoustic disturbances—incorporating spatial dynamics and movement behaviour into models can improve predictive accuracy. Biotic interactions often play a crucial role in shaping population‐ and community‐level responses. For example, disease transmission relies heavily on interactions between individuals, while habitat degradation may trigger density‐dependent effects. As climate and land‐use change accelerate (IPBES 2019), models are increasingly required to predict outcomes under novel environmental conditions. Integrating genetic and evolutionary processes allows simulated populations to adapt to these new scenarios, especially when combined with other mechanisms such as movement and energetics.

Because many indirect stressors affect individual physiology, incorporating energetic processes offers a valuable approach for scaling the impacts of indirect or multiple stressors from the individual level up to populations and communities (Figure 1, Box 1). Energetics, the way energy is acquired and allocated for physiological processes, is a unifying mechanism across all organisms. By capturing the underlying processes of energy flow, energetic models can make useful predictions about organism responses to environmental changes such as resource availability, temperature and stress (e.g., Molnár et al. 2010; Harwood et al. 2020), providing an explanatory framework linking physiology and ecology. Modelling energetics can thus provide a greater understanding of how, when and why animal populations are impacted by indirect or multiple stressors. This consideration is particularly important in the context of ongoing climate change and increasing human activity (IPBES 2019).

Energetic models can vary considerably in their structure, complexity and data needs. For example, ‘traditional’ bioenergetic models (*sensu* Nisbet et al. 2012) are conceptually intuitive, with clear links between model parameters and empirical processes. However, these models are often parameter‐rich and less generalisable across species. In contrast, Dynamic Energy Budget (DEB) theory (Kooijman 2010) applies universally to all living animals, from bacteria to whales. DEB theory is grounded in the fundamental principles of mass‐energy balance that scale across levels of biological organisation, and its ‘standard DEB animal model’ requires fewer parameters than traditional bioenergetic models (Sousa et al. 2010). However, in contrast to the traditional bioenergetic models (Nisbet et al. 2012), few DEB parameters can be directly related to empirical data.

Both traditional and DEB‐based energetic models have been incorporated into a range of individual‐ (e.g., McHuron et al. 2021), population‐ (e.g., Rademaker et al. 2024) and community‐based models (e.g., Szangolies et al. 2024). Given the common challenge of data availability, the DEB approach may be more attainable than traditional bioenergetic approaches that require detailed energetic data that may not be readily available. However, traditional bioenergetic approaches can utilise data from proxy species and allometric relationships, which may be adequate for the research purpose. Finally, traditional bioenergetic models may be more readily linked with individual movement behaviour than DEB models (but see Malishev et al. 2018), which can provide valuable insight into disturbance impacts with spatially varying levels of exposure.

## 2 Approaches to Modelling the Effects of Disturbance on Wildlife Populations

Here, we provide an overview of the key approaches for understanding and predicting the impacts of disturbance on individuals, populations and communities. We use a broad definition of human disturbance, wherein we include natural processes that can be exacerbated by human activity (e.g., climate variation, disease, wildfire). We have categorised the modelling approaches into four sections based on the ecological scale at which disturbance is usually modelled for each approach. These have been broadly broken down into the individual, population, community, or geographical range scales. We recognise from the outset that some modelling traditions include or link components from multiple scales and that some approaches represent broad categories of model families, while others are specific to a single model or method. Reference to more in‐depth reviews of specific approaches is provided in the Supporting Information.

### 2.1 Individually‐Focused Dynamics

The approaches below typically allow individuals (or groups/subsets of individuals, without a strict size threshold) to vary within a population in terms of a variety of traits related to behaviour, genetics or energetics. Accounting for such variation may be more realistic than those that assume all individuals are identical (Denny 2017); which can improve predictions of population dynamics (Gerber 2006).

### 2.2 Individual‐Based Models

Individual‐based models (IBMs or agent‐based models) are a broad class of simulation models that depict relevant processes at the individual or group level (the agent). Population‐level properties (e.g., population growth rate) emerge from the behaviour of, and interactions among, discrete agents through time. This key property makes IBMs particularly useful when intraspecific trait variation, local interactions, adaptive behaviour, or heterogeneous environments are assumed to influence population level responses to disturbance (Chevy et al. 2025; DeAngelis and Grimm 2014), as well as for small populations (Caughley 1994).

Stochastic dynamic programming is an optimisation method frequently used to identify optimal decisions in dynamic and uncertain systems (Marescot et al. 2013). In behavioural ecology, these models are built with the assumption that individuals should act to maximise some future expected reward, such as Darwinian fitness (Houston et al. 1988; Mangel and Clark 1988). Future expectations may depend upon intrinsic (e.g., body condition) and/or extrinsic states (e.g., location), providing state‐dependent optimal decisions (e.g., whether to reproduce or where to move) that can then be used within an IBM framework to predict individual‐ and population‐level responses to disturbance.

Due to their flexibility, IBMs are frequently used to assess the population impacts of disturbance, either as a stand‐alone method or in conjunction with other approaches described throughout this section (Figure 4). Such applications include investigating the effects of climate change and habitat connectivity (e.g., Andersen et al. 2022; Johnson et al. 2024), toxicant exposure (e.g., Hall et al. 2018), and acoustic disturbance (e.g., McHuron et al. 2021) on population dynamics, where individual exposure was known or assumed to vary. IBMs are also useful tools when little is known about populations since models can still be developed that provide guidance for data‐limited species, as demonstrated in our mink case study.

### 2.3 Cell‐Lattice Models

While not technically focused on individuals, cell‐lattice models are grid‐based spatial models that treat individuals within the cell as a subpopulation with a spatially explicit set of state variables and rules. Discrete cells can be characterised by variation in important landscape attributes, such as habitat type, food availability or predation risk, that influence demographic rates. Dispersal between adjacent cells is used to depict simple patterns of redistribution by the fraction of the subpopulations arising from neighbouring cells. Cell‐lattice models are a computationally simple way to evaluate the influence of habitat arrangement, mobility and behavioural decision‐making on rates of resource gain and mortality risk among subpopulations occurring in different cells at a given point in time (Tonini et al. 2014). Because of their inherent spatial nature, cell‐lattice models are particularly relevant for applications involving movement barriers (e.g., road infrastructure; Holdo et al. 2011), invasive species spread (Tonini et al. 2014) and disease spread (Jeltsch et al. 1997).

### 2.4 Population Dynamics

Modelling human impacts at the population level has a long history (Boyce 1992; Lande 1993). These approaches directly link human disturbances to population viability by quantifying how shifts in key demographic processes (Morris and Doak 2002), such as survival and reproduction, influence population growth and structure (Caswell 2000).

### 2.5 Structured Population Models

Structured population models describe the state‐dependent dynamics of a given population by linking vital rates with population processes in discrete time. Matrix population models and integral projection models are structured population models that represent population dynamics in discrete time but differ by structuring states in discrete (e.g., developmental stage) or continuous (e.g., size) stages, respectively.

Matrix population models provide a relatively straightforward approach for evaluating population‐level disturbance effects, particularly when disturbances differentially impact discrete life cycle stages, such as assessing the impacts of culling on specific age classes (e.g., Davis 2022). In contrast, integral projection models are more suitable for assessing the impacts of disturbance that vary with continuous traits, such as the impacts of size‐selective harvesting (e.g., Stubberud et al. 2019). Integral projection models can provide insight into ecological patterns including population dynamics, species distributions, or life‐history strategies by integrating vital rates with environmental covariates through the use of regression models (Merow et al. 2014).

To gain mechanistic insights into ecological patterns under disturbance and improve predictions to novel environmental conditions, DEB theory has been integrated into structured population models (Billoir et al. 2007; Smallegange et al. 2017; Thunell et al. 2023). Mechanistic applications that incorporate energetics allow for the investigation of ecological and evolutionary patterns from an energy budget perspective, such as sensitivity to shifts in environmental variability (Smallegange et al. 2020; Rademaker et al. 2024) or the eco‐evolutionary consequences of climate change for populations (Thunell et al. 2023).

### 2.6 Hierarchical Models

Hierarchical models are highly flexible statistical models designed to handle data that are nested or from disparate sources. Model‐based data integration approaches, such as combining quantitative and qualitative trends within a unified framework (e.g., Johnson et al. 2023), are increasingly used to improve inference across heterogeneous data sources. We discuss two types of hierarchical models relevant to disturbance ecology: state‐space models and integrated population models.

State‐space models are used to explicitly separate the underlying ecological process (the true, unobserved state; e.g., actual population abundance) from the observation process (measurements; e.g., annual census count). This separation of ecological and observation processes enables independent estimation of uncertainties arising from biological stochasticity and sampling‐related measurement errors—reducing bias in parameter estimates compared to models that account for only one source of uncertainty (Auger‐Méthé et al. 2021). As a result, this approach is particularly useful for handling noisy time‐series data from disparate sources. This is evidenced, for example, through the application of state‐space models to time‐series data on host‐parasitoid dynamics (e.g., Karban and De Valpine 2010) and population abundance (e.g., Westcott et al. 2018).

Integrated population models (IPMs) use a single statistical framework to combine population counts and demographic data, enabling inference on population dynamics. IPMs are often implemented using state‐space models to account for parameter uncertainty, with a structured population model at their core to track changes in population size. The multimodal structure, while powerful, means that IPMs can be complex to implement and interpret. Nonetheless, IPMs can help reduce uncertainty in parameter estimates, estimate confounded or hidden parameters, and disentangle sources of uncertainty when forecasting population trajectories (Schaub and Abadi 2011). Because of their ability to handle multiple data sources and reduce uncertainty, IPMs have been applied to predict the population consequences of a range of disturbances (e.g., Gamelon et al. 2023; Oppel et al. 2022). Some work has also extended these models to multiple species (Péron and Koons 2012; Quéroué et al. 2021).

### 2.7 Continuous‐Time Population Models

Since some ecological processes, such as births, deaths and migration, can occur continuously rather than at fixed intervals, continuous‐time models can provide more realistic insights into population dynamics for some species. Here, we discuss two types of continuous‐time models that are used in disturbance ecology: partial differential equations and physiologically structured population models. Both approaches are mathematically complex and computationally expensive, but have comparatively lower data requirements than other approaches discussed.

Partial differential equations are a class of mathematical equations used to describe systems where variables change continuously over both time and space. These equations express relationships between the rates of change of these variables with respect to time, spatial dimensions, or both. As such, partial differential equations are particularly useful for processes that have a strong spatio‐temporal dependence (e.g., dispersal, ecological invasions), leading to applications modelling climate change (Chhaytle et al. 2023; Goel et al. 2020), invasive species (Laplanche et al. 2018) and pest control (Banks et al. 2020).

Physiologically structured population models can describe a population's demography using physiological principles, focusing on interactions between physiology, population structure and the environment (Metz and Diekmann 1986; de Roos 1997). This approach can provide in‐depth mechanistic insights into the impacts of disturbances relating to physiology, such as the population‐level impacts of food limitation (Hin et al. 2019) or environmental stress (Silva et al. 2020), and is particularly useful for equilibrium analyses. A computational approach (*PSPManalysis*; de Roos 2021) exists that merges discrete‐ and continuous‐time approaches, providing typical matrix model outcomes (e.g., population growth rate) that may be useful for management applications.

### 2.8 Metapopulation Models

Metapopulation models represent the multiscale dynamics of species inhabiting discrete habitat patches, where populations face measurable extinction risk, can recolonise after local extinction and experience asynchronous local population dynamics. Since these models are unified by a common theoretical framework rather than a standardised methodological approach, there is considerable diversity in modelling techniques available that may represent time and space differently (Ovaskainen and Hanski 2001; Bond et al. 2023; Souto‐Veiga et al. 2024). Dynamics may be represented using IBMs (e.g., Radchuk et al. 2013) and matrix population models (e.g., Takashina 2016), among others (e.g., Brandell et al. 2021; Zhang et al. 2021). Because connectivity directly influences recolonisation success and local persistence, these models are well positioned to quantify the role of dispersal corridors, stepping‐stone habitats and landscape permeability in maintaining metapopulation stability.

### 2.9 Range Dynamics

From a disturbance ecology perspective, studying range dynamics can tell us where populations may be at greater risk of exposure to stressors, highlight potential areas of refuge and identify key habitat requirements for a population to persist. This information can be used to prioritise, for example, areas for protection or management. While some of the approaches above (e.g., IBMs) are capable of predicting range dynamics, the methods below are primarily designed to address range dynamics, with demographic processes treated as secondary factors or omitted entirely.

### 2.10 Correlative Species Distribution Models

Correlative species distribution models (SDMs) identify statistical relationships between species occurrence or abundance and spatio‐temporal patterns of environmental variation to explain or predict species distributions (Elith and Leathwick 2009). Disturbance can be incorporated as a predictor variable or effects can be inferred based on spatial or temporal shifts in habitat suitability. These models are also frequently integrated as a spatial layer for other modelling approaches, such as connectivity models (e.g., Rezaei et al. 2022) and IBMs (e.g., Andersen et al. 2022; Jordt et al. 2016), providing boundaries for movement or dispersal (Figure 4). However, interactions among stressors, discrepancies in spatio‐temporal scales of impact and the correlative nature of SDMs can limit their ability to inform policy or management, particularly when predicting under novel conditions (e.g., Lee‐Yaw et al. 2022; Sirén et al. 2025). Despite transferability and extrapolation concerns, the data required for SDMs are often readily available and alternative methods may be unfeasible, making them the best option available for some populations.

Machine learning algorithms are computational approaches that learn patterns from large and complex datasets capturing non‐linearities and complex interactions between variables to generate accurate predictive models (Pichler and Hartig 2023). In addition to their use for SDMs, machine learning models have been used to understand the impact of disturbance on animal behaviour (e.g., Fardell et al. 2021; Tédonzong et al. 2020). In this way, machine learning models can support other modelling methods. For example, they can be used to estimate parameters in mechanistic models (Noordijk et al. 2024).

### 2.11 Process‐Explicit Range Models

Process‐explicit range models extend correlative SDMs to explicitly model the underlying processes that drive population dynamics, such as physiology, dispersal, demography and evolution (Briscoe et al. 2019). There is a broad range of modelling approaches that can be categorised as process‐explicit range models (e.g., occupancy models, coupled SDM‐population models, eco‐physiological models), which differ in the biological processes that they include (reviewed in Briscoe et al. 2019). These models are often assumed to provide more accurate range prediction when extrapolating to novel conditions than correlative approaches (Evans et al. 2015; but see Uribe‐Rivera et al. 2023). As such, eco‐physiological range models are frequently used for predicting range shifts under climate change (e.g., Santika et al. 2014), since future climate conditions are expected to be outside the range of conditions already experienced by a population (Briscoe et al. 2023). Demographic‐based range models have also been used to link correlative SDMs with population or dispersal models to gain greater mechanistic insights into disturbances impacts (e.g., Bastos et al. 2016; Pratzer et al. 2023). These approaches are, however, more complex and data hungry than correlative SDMs.

### 2.12 Community and Ecosystem Dynamics

While ecological models often focus on a single species, unintended management outcomes can result when species are viewed in isolation (Buckley and Han 2014). Models that explicitly consider the interactions and feedback among species can help better inform population‐level responses to disturbance and management. Community and ecosystem models vary substantially in their complexity, from simple food webs (e.g., Varriale and Gomes 1998) to complex end‐to‐end ecosystem models (e.g., Fulton 2010). Here, we focus on metacommunity and food web models as these approaches are more frequently applied to disturbance studies, though their application is still relatively rare.

### 2.13 Metacommunity Models

Metacommunity models are analogous to metapopulation models, representing multiscale dynamics of multiple species in discrete habitat patches, in the context of extinction and dispersal, with additional consideration for competition and trophic dynamics. Patterns in local extinctions versus regional survival are central to these models and are driven by processes such as environmental filtering, biotic interactions, dispersal and drift (Chase et al. 2020; Lerch et al. 2023). Despite being rarely used to study disturbances, metacommunity models can be useful for understanding biodiversity patterns and community assembly across landscapes in the face of disturbance (Dugger et al. 2011).

### 2.14 Food Web Models

Food web models aim to represent the demographic impact and rates of transfer of material or energy between different elements of the community matrix as a result of trophic interactions such as predation, parasitism, or mutualism (e.g., Baudrot et al. 2020). These models encompass a wide variety of computational approaches, ranging from partial differential equations (e.g., Lusardi et al. 2024) to individual‐based or cell‐lattice models (e.g., Fryxell et al. 2020). Community structure, behavioural details (e.g., decision‐making, mobility and cognition), sources of heterogeneity affecting interaction rates, and landscape configuration are often key components influencing model outcomes. The response to disturbance in food web models, such as that caused by invasive species or habitat loss (e.g., Roemer et al. 2002), is often determined through impacts on community structure or changes in the functional relationships between community components.

## 3 Case‐Studies

To illustrate how modelling approaches can be selected for a given application, we highlight two case studies: (1) the ever‐abundant red fox and (2) the critically endangered European mink. The red fox is a well‐studied species that has been modelled extensively to address a range of management purposes. The European mink, on the other hand, is a data‐limited species of high conservation concern, with only one modelling application. Through this endeavour, we hope to illustrate some of the decisions that are made in the model development process, how others have overcome the limitations of different approaches, and how mechanistic pathways can be used to help address data scarcity challenges.

### 3.1 Case‐Study 1: Red Fox

**FIGURE 5:**
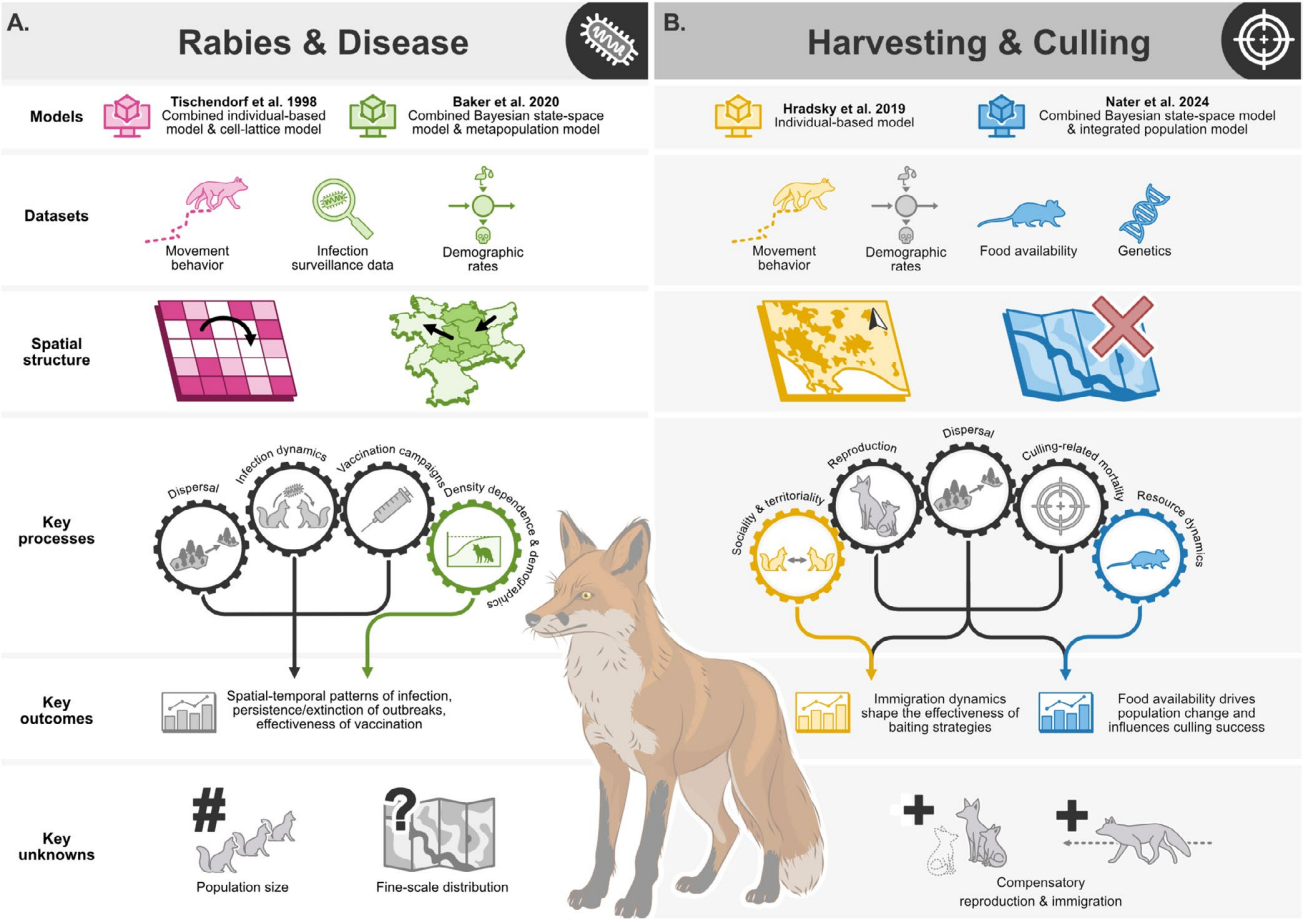
Summary of the red fox (Vulpes vulpes) case‐study model comparisons for (A) rabies and (B) culling, highlighting the primary data types used, the spatial structure, key processes represented, model outcomes and the key unknowns when modelling red fox population dynamics. Each model is colour coded to indicate which elements are specific to a single model (coloured) or shared between both models (grey).

Disturbances of wildlife populations are often unintentional, occurring as a by‐product of other human activities. In some cases, however, they intentionally result from targeted management actions that occur in isolation or conjunction with other disturbances. This can be exemplified well with the red fox, a small carnivore that has a wide distribution across the northern hemisphere (Box 2). Foxes are often perceived as a nuisance species, are potential vectors of zoonotic disease, but also play important ecological roles (e.g., prey regulation). As a relatively well‐studied species of high management interest, a range of modelling approaches have been used to address disturbances in red fox populations. Here, we provide a brief overview of some of these models, focusing on two disturbances where multiple methods have been used to address similar questions, namely rabies and culling (Figure 5).

BOX 2 **Ecology of the red fox.**

**Distribution and conservation status:** The red fox has the largest geographical range of all members of the order Carnivora. It is widespread throughout the northern hemisphere, from the Arctic Circle to southern North America, Europe, North Africa, the Asian steppes, India and Japan. Introduced populations also persist in Australia where they have caused significant damage to native ecosystems. The species is listed as Least Concern (LC) on the IUCN Red List (Hoffmann and Sillero‐Zubiri 2021).

**Ecology:** Red foxes can be found in habitats as diverse as tundra, deserts, mountains (up to 4500 m), forests and urban areas. Foxes are opportunistic omnivores and scavengers with highly plastic diets that vary according to the availability of food resources. Their diet may consist of mammals (voles, rabbits, young hares or lambs), ground‐nesting birds, poultry, invertebrates, fruit and food waste, to varying degrees. Females become sexually mature around 10 months old and generally give birth to a litter of 4–6 young per year. Red fox densities vary widely, from as low as 0.02 ind/km^2^ in rural areas (Meia 1994), up to 30 ind/km^2^ in urban areas where there is an oversupply of food (Harris and Rayner 1986). It can be solitary at low densities but also forms social groups (Macdonald 1981).

**Main threats and management actions:** Threats to this species from humans include habitat degradation, loss and fragmentation in certain areas, exploitation and persecution (Hoffmann and Sillero‐Zubiri 2021). Hunting and trapping are widespread in most areas, with large kill bags. Hunting is mostly seen as sport, while trapping and regulatory shooting aim to reduce population size and depredation, but there is increasing debate about whether fox control is achieving its goals. While no longer considered a concern, red fox populations have historically been impacted by vulpine rabies epidemics.

### 3.2 Rabies

Rabies, a zoonotic disease, is often viewed as a natural feature in the environment. However, its dynamics, along with those of other diseases, can be shaped by human influences, including spill‐over from domestic species, shifts in wildlife densities driven by urbanisation and changes in behaviour or ranges associated with climate or land‐use change. Rabies has been extensively studied in red foxes, which serve as critical hosts and vectors for specific strains of the disease. Although now eradicated in many regions, rabies remains a valuable case‐study due to the diverse modelling efforts it has inspired, offering opportunities to compare alternative approaches that may be applied to other diseases of interest.

Here, we compare two multimodal methods for modelling rabies dynamics: (1) an IBM with a cell‐lattice framework (Tischendorf et al. 1998) and (2) a combined Bayesian state‐space and metapopulation model (Baker et al. 2020) (Figure 5). Tischendorf et al. (1998) employed spatially explicit grid cells to simulate localised interactions, transmission heterogeneity and clustering effects in highly immunised fox populations, offering insights into fine‐scale processes and enabling targeted interventions. In contrast, Baker et al. (2020) used three decades of rabies case data to assess regional spatial coupling, density‐dependent dynamics and localised transmission to capture broader trends and demographic influences of rabies on fox populations. Despite differing in the specific research aims, both approaches incorporated the majority of the key processes that may be important for modelling the impact of disease on animal populations (Figure 2) and provided complementary insights into rabies dynamics by focusing on different scales and mechanisms of disease spread.

Both models incorporated seasonality and dispersal, essential for capturing temporal variations in long‐distance transmission and changes in the number of susceptible individuals. However, their use of empirical data differed. Tischendorf et al. (1998) relied on literature‐derived movement patterns, while Baker et al. (2020) used Bayesian approaches to estimate dispersal rates from observed rabies cases. Tischendorf et al. (1998) also used theoretical landscapes, while Baker et al. (2020) represented regions as five German states between which dispersal could occur. Notably, only Baker et al. (2020) explicitly integrated density dependence (represented as declines in survival and reproduction as metapopulations approach carrying capacity) and demographic processes, critical for realistic modelling of contact rates. In Tischendorf et al. (1998), these processes were somewhat implicitly represented through the number of occupied cells, mortality rates due to infection and dispersal rates of subadult foxes.

The use of multiple modelling approaches in both cases demonstrates the benefits of model integration for addressing disease dynamics, while managing the trade‐offs inherent with modelling complex systems (Figure 4). For example, both approaches captured spatial elements of rabies dynamics and tracked disease progression over time, demonstrating that different approaches can achieve similar goals. Both approaches also emphasised the importance of spatio‐temporal processes in understanding rabies dynamics. Both studies found that incorporating dispersal‐mediated transmission across habitat regions was important to reproduce key empirical patterns. Despite these strengths, both models faced challenges with missing data, such as fox population size and fine‐scale distribution of foxes and vaccination campaigns, which would have improved model calibration and validation. Ultimately, each approach underscored the necessity of accounting for local interactions and spatial heterogeneity to model the complex fox‐rabies system, strengthening the conclusions despite data limitations.

### 3.3 Culling

Foxes are subject to lethal predator control through harvesting, fertility control and poisoning due to their perceived negative impacts on wildlife, livestock and human health, with the aim to limit depredation impacts and/or reduce disease spread (Hoffmann and Sillero‐Zubiri 2021). However, the impact of culling on fox population dynamics remains unclear due to a lack of evidence of potential compensatory mechanisms (Lieury et al. 2015). Beyond foxes, understanding the effectiveness of predator control remains a key issue in conservation management.

Here, we discuss two approaches used to evaluate the impact of culling on fox population dynamics: (1) a spatially explicit IBM (Hradsky et al. 2019) and (2) a Bayesian state‐space IPM (Nater et al. 2024) (Figure 5). These two approaches had different management purposes and thus required different data and considered different processes. Hradsky et al. (2019) focused on the impact of poisoning to evaluate population responses to diverse baiting designs at scales relevant to management, while Nater et al. (2024) assessed the impact of harvesting on vital rates, population structure and rate of population change in an expanding fox population. Despite their differences, both modelling applications included the majority of the key processes that are likely important for predicting the impacts of exploitation on animal populations (Figure 2).

To evaluate and plan effective fox baiting programs, Hradsky et al. (2019) used customisable habitat‐cells to specify habitat patches and indicate the location and type of bait stations. The model used a relatively fine temporal scale that allowed for fox sociality and territoriality to be incorporated. At each time step, foxes could disperse and depending on their sex and social status, join a fox‐family and reproduce. In contrast, Nater et al. (2024) used a non‐spatial, female only model to understand the drivers of fox population dynamics. Their model was built on an annual time step, during which the population changes in response to natural mortality, harvesting, immigration and reproduction. The impact of seasonal and inter‐annual changes in food availability on local demography and immigration rates were also investigated.

To investigate the effects of culling in their respective contexts, the authors utilised different data and evaluated their models in different ways. Hradsky et al. (2019) parameterised their IBM using site‐specific data from the literature, including population density and dispersal distances. Their model was applied to four case studies and model outputs were validated against individual‐ and population‐level empirical estimates. Nater et al. (2024), on the other hand, used a range of disparate data streams to estimate age‐specific demographic rates (number, age and reproductive status of harvested foxes), reproductive rates (placental scar data and opportunistic pup counts from hunters and camera traps), and immigration rates (genetic data). Data on food availability (rodent abundances and reindeer carcasses) at different spatial and temporal scales were also used to infer natural mortality and immigration. The IPM was then evaluated by comparing model predictions with genetic data on emigration.

Overall, both approaches provided complementary insights on the impact of culling on fox populations. Hradsky et al. (2019) showed that fox density is more sensitive to the frequency of baiting than the spatial density of baits, due to the recruitment of individuals from neighbouring patches. In contrast, Nater et al. (2024) identified the key drivers of year‐to‐year population change, highlighting the interactive role of food availability, showing that harvesting is more efficient when it coincides with low rodent abundance. Both studies highlighted the importance of better understanding density‐dependent and compensatory fecundity and immigration, which appear to be key drivers of fox population dynamics. Potential immigration‐mediated compensation for intentional mortality has rarely been investigated due to the lack of data on dispersal. In this regard, the IPM developed by Nater et al. (2024) shows a very promising use of genetic data for estimating migration rates.

### 3.4 Case‐Study 2: European Mink

Many species are subject to data limitation challenges, making it difficult to assess conservation status and to identify the associated drivers of population decline. Regardless, management decisions are needed, often at timescales that are much shorter (years) than it takes to amass the data to conduct robust analyses on population dynamics (decades). Rare species present a particular challenge because their scarcity makes data inherently difficult to collect, while also being at high risk of extinction (Davidson et al. 2009). The European mink, a mustelid that has been extirpated across much of its historic range, is one example of this situation. Remaining local populations are critically endangered and active intervention to promote recovery is ongoing (Box 3).

BOX 3 **Ecology of the European mink.**

**Distribution and Conservation Status:** The European mink is the most endangered mammal in Europe and is classified as Critically Endangered (CR) on the IUCN Red List (Maran et al. 2016). Over the last 150 years, the species has declined by more than 90% and has been extirpated or severely depleted across most of its former range. The current range of the endemic wild population consists of a few isolated fragments in northern Spain and western France, the Danube delta in Romania, Ukraine and Russia (Maran et al. 2016).

**Ecology:** European mink are small semi‐aquatic mustelid carnivores (males: 0.5–1.5 kg, females: 0.3–0.7 kg). They inhabit densely vegetated banks of rivers, streams and lakes with stagnant or slow‐flowing water across a variety of landscapes (forests, agricultural, hedgerows, marshes, polders, etc.), using underground burrows or dense vegetation for resting and reproduction. Diets primarily consist of amphibians, crustaceans (crayfish), fish, small mammals (rats and voles), birds and, to a lesser extent, insects and eggs (Palazón et al. 2004; Libois 2001). Predators include the red fox, dogs and raptors (Maran et al. 2017; Põdra 2021). Females reach sexual maturity at 11 months and give birth to a litter of 2–7 young per year. Longevity in the wild has been reported to be up to 5 years (Mañas et al. 2016).

**Main causes of decline and management actions:** The main hypothesised threats contributing to the current decline are (1) habitat loss and fragmentation of wetlands, (2) road mortality, (3) harvesting and (4) the impact of the invasive American mink through interference competition. The species is protected by law in Europe. In an effort to recover European mink populations, captive breeding and reintroduction programs have been implemented in Spain (Gomez 2018; Maran et al. 2017; Põdra 2021), Estonia (Maran et al. 2017) and France (Direction Régionale de l'Environnement, de l'Aménagement et du Logement (DREAL) 2021).

**Data availability statement:** Across the three extant populations, available data include presence data, home range size estimates, diet information in the presence and absence of invasive competitors, habitat requirements, trait data, behavioural responses to humans and post‐release survival estimates for captive bred individuals.

For understanding disturbance impacts and informing management decisions for European mink, correlative SDMs are an obvious first choice as presence data exist and remote sensing and climate modelling make it possible to include dynamic and disturbance‐relevant predictor variables. SDMs have been developed to predict habitat suitability for European mink (and American mink, *Neovison vison* , an invasive competitor) in Spain under historical conditions and various socioeconomic and emissions pathways (Goicolea et al. 2023). Spatial maps produced from SDMs can help identify areas for protection, restoration and captive release, and illustrate changes in habitat suitability and interspecific overlap under climate change. The latter relies on assumptions that correlative relationships remain unchanged in time, accurately represent species requirements, and hold when extrapolated outside the range of input data. However, in many instances, these assumptions are likely to be violated.

**FIGURE 6:**
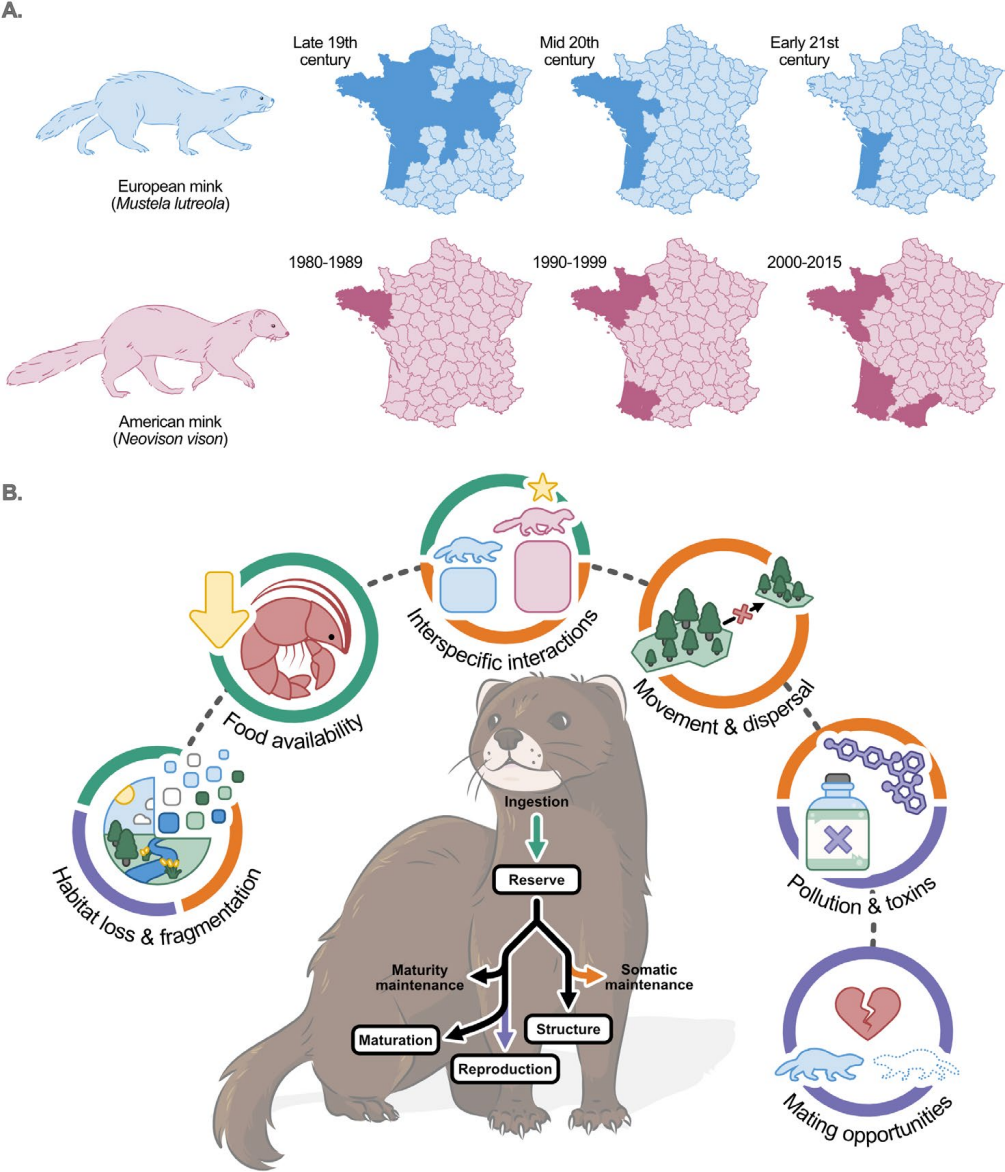
Schematic for the European mink (Mustela lutreola) case study, highlighting the (A) range contraction of European mink and concurrent expansion of invasive American mink (Neovison vison) in France, and (B) potential energetic impacts of key threats to the European mink, illustrated using dynamic energy budget (DEB) theory. Threats are colour coded to indicate how they affect the energy budget: Green‐via ingestion (e.g., reduced food intake), orange‐via somatic maintenance (e.g., increased energy expenditure) and purple‐reproduction (e.g., reproductive impairment). The white boxes over the mink refer to key energy stores used in DEB theory. Maturity and somatic maintenance are energetic costs.

Many of the potential causes of the decline in the European mink could have strong impacts on energy balance (Figure 6, Box 3). Energetic modelling approaches are well suited to data‐limited species because many energetic processes scale allometrically or are evolutionarily conserved (McGrosky and Pontzer 2023; Kooijman and Augustine 2022), allowing models to be parameterised in the absence of species‐specific data. In addition, while energetic measurements from data‐limited populations may be difficult to obtain, data collected from proxy species or animals managed in human care may be more readily available. For example, metabolism and reproductive energetics have been measured in American mink and other terrestrial mustelids (e.g., Iversen 1972; Wamberg and Tauson 1998; Chappell et al. 2013), while data relevant to energetic models have been collected from European mink in captive breeding programmes (Kiik et al. 2017). These data can thus inform the energetic requirements and challenges of the European mink.

The foundation of DEB in first principles allows for the transfer of information across species by leveraging shared biological principles, allowing for predictions even for species with limited data (Lika et al. 2011). The standardised model structure also facilitates the application of existing models to new species, particularly to closely related species, as could be the case with American and European mink (Desforges et al. 2017). However, extending DEB models into spatially explicit models can be challenging. Leveraging existing data, allometric relationships, and data from proxy species within a traditional bioenergetic model may simplify extension to a spatially explicit framework.

For the European mink, energetic models could be used to investigate the effects of habitat loss and fragmentation, including reduced prey availability, interspecific competition, altered activity budgets and antagonistic interactions with American mink (Figure 6). Other disturbances indirectly related to energetics could be incorporated, such as pollution or reduced mating opportunities, to understand synergistic impacts. Such models could help identify thresholds at which resource scarcity begins to negatively affect individual survival, assess the long‐term impacts of disturbances and quantify the impacts of stressor removal (e.g., eradication of American mink). By combining energetic models with individual‐based movement models and habitat suitability maps (from SDMs), spatially explicit energetic models could be used to evaluate the potential outcomes of management decisions. For example, this approach, coupled with targeted surveys, would allow for the identification of suitable areas to release captive‐bred individuals based on resource availability. It could also inform habitat restoration efforts by highlighting areas where interventions would likely have the greatest impact on the species' recovery.

### 3.5 Summary of Case‐Studies

These case‐studies have exemplified the diversity and complementarity of modelling approaches for predicting the population consequences of human disturbance. Our fox case study demonstrated that IBMs are particularly well suited for assessing human impacts on species with extensive ecological knowledge available, thus enabling detailed simulations of individual behaviour and interactions (Figure 4). Our European mink study underscored the potential for energetics modelling to overcome data limitation issues that are common for species of conservation concern. Although particularly suited to data‐limited species, energetics approaches are also useful for other species exposed to indirect stressors (Johnson et al. 2024). Despite the breadth of existing research, a notable gap exists in the red fox literature regarding the integration of energetics with disturbance modelling. Given the growing body of empirical research on red fox energetics, there is an exciting opportunity to link disturbances to population and community dynamics through changes in individual energetics and vital rates. Doing so could quantify the energetic costs of disturbances (e.g., by measuring the energetic demands imposed by habitat fragmentation or reduced prey availability) or predict long‐term population or community consequences by linking individual energy budgets to reproduction, survival rates and interspecific interactions.

## 4 Discussion

An impressive variety of quantitative modelling approaches are being used to understand and predict wildlife responses to human disturbance—and even to address the same conservation or management problems, as exemplified by our red fox case study. Understandably, it may be daunting for someone who is not familiar with modelling, or who is only familiar with a specific family of models, to decide on which modelling approach(es) to use. We hope that this manuscript can provide guidance and broaden horizons for those wishing to model the impacts of human disturbance on wildlife. Broadening our perspective of what constitutes disturbance can open ecologists up to other research areas and approaches. For example, some ecologists may not consider disease to be a human‐mediated disturbance, since it is a part of the evolutionary history of most organisms, but it is also affected by human‐mediated environmental change. Approaches used to model disease impacts, and their associated considerations, may thus provide insights into the modelling of other disturbances that may act via similar mechanisms. A broad perspective can also expose ecologists to some of the ways that modelling approaches can be integrated to overcome the limitations of the different singular approaches.

It is interesting how different processes are being pulled into models to address different disturbances. This was illustrated well for red foxes, where dispersal and immigration were explicitly incorporated when assessing the impacts of regulatory management, since these processes appear to drive the difficulties in regulating red fox abundance. Similarly, spatial dynamics, movement and dispersal were key processes included for predicting disease transmission, because movement of infected individuals drives disease spread. The differences in how processes were modelled may reflect differences in data availability, modeller experience and/or the research question. Nonetheless, this diversity of representations demonstrates the importance of these ecological processes for understanding population dynamics and highlights some strategies for including these processes given varying constraints.

Despite the diverse representation of ecological processes in modelling applications, there remains an opportunity to incorporate other processes that impact population responses to human disturbance, including eco‐evolutionary feedbacks and sociological processes. Since evolutionary changes following disturbance may enable populations to adapt to disturbances, such as increasing temperatures, eco‐evolutionary feedbacks play a crucial role in the long‐term responses of populations to disturbance (Loeuille 2019) (Figure 2). Social dynamics can also play a key role in population dynamics, and although they are sometimes considered for social wildlife (e.g., Brandell et al. 2021; Grente et al. 2024), the human element is often neglected. Not only are human disturbances inherently driven by human behaviour, but so are the perceptions of management actions (Bro‐Jørgensen et al. 2019). Such interdisciplinary approaches are already well developed (e.g., Dobson et al. 2019) but greater uptake in disturbance ecology modelling could improve conservation and regulatory management outcomes.

We were pleased to see increasing use of energetic modelling applications across a range of quantitative approaches. Since energetics act in a summative way and many non‐lethal disturbances have impacts on energy acquisition or use, energetic models can help us better understand the impacts of multiple stressors on wildlife populations. Subsequently, the inclusion of energetic mechanisms represents an important avenue to scale individual‐level responses to population‐ and community‐level impacts. Perhaps most importantly, energetic models may offer the only viable way both scientists and decision‐makers can anticipate the impact of demographic responses to complex patterns of global climate change that will surely continue for the foreseeable future. If we do not accommodate changes in behaviour, space use and demography that will accompany the relentless change in climatic drivers, even the best of models will only have value for understanding the past rather than predicting the future.

Despite the developments and integrations of modelling approaches, models can only ever be a simplified representation of natural systems. All modelling approaches rely on assumptions, imperfect data and simplifications of the processes they aim to represent. Consequently, there is a great deal of uncertainty associated with input parameters and data, model structure (Refsgaard et al. 2006), and resulting model predictions (Rounsevell et al. 2021). Several approaches have been developed to minimise or quantify the level of data or model uncertainty. For example, sensitivity analysis aims to determine the influence of uncertain parameters in model outputs, which can highlight priority areas for data collection or model development (Cariboni et al. 2007). Alternatively, ensemble modelling aims to reduce model uncertainty by combining predictions from multiple models. This approach has become more common for species distribution modelling (Hao et al. 2020) but has received criticisms due to the ‘smoothing out’ of model outputs. Challenging models with different assumptions against each other (i.e., robustness analysis (Levins 1966) or model intercomparison) is another approach to quantifying uncertainty in model outputs, as well as investigating the influence of different model structures.

Quantifying uncertainty brings about its own challenges. When providing predictions to managers, it is important to highlight the degree of uncertainty in model predictions; yet uncertainty can make it more difficult for managers to make decisions. This makes knowledge transfer crucial, as stakeholders are able to conceptualise uncertainty in model outputs and make informed decisions when clearly communicated (Mahevas and Sigrid 2024). Nonetheless, the acceptance of modelling tools as decision support tools depends on whether different stakeholders agree with the representation of the system and their understanding of its components. For this purpose, participatory modelling is a widely used approach that aims to increase and share knowledge of a system and its dynamics under different conditions and to anticipate the impact of management actions to support decision‐making (Voinov and Bousquet 2010). However, the involvement of stakeholders is no guarantee of the appropriation of model results, especially if their participation is limited.

## 5 Conclusion

The diverse methodological toolkit available highlights the adaptability of modelling approaches to address specific stressor types and questions for wildlife conservation and management. Here, we have provided an overview of the key modelling options available for predicting population responses to human disturbance, indicating how different models can be combined to leverage the strengths of alternative approaches. We also highlight the important role that energetics plays in predicting the impacts of indirect stressors on population dynamics and suggest other areas of development in modelling the complexities of indirect or multiple stressors, such as eco‐evolutionary and sociological mechanisms. While not an exhaustive list of all of the available approaches, we argue that we capture the key diversity of approaches currently used for predicting the impacts of human disturbance on animal populations and communities. We hope new studies will consider alternative approaches or integrations and identify the processes that should be incorporated for assessing the specific disturbances impacting a given study system. Throughout, we have illustrated the value of integrating different modelling approaches to address population‐ and community‐level consequences of disturbance, demonstrating that science is stronger with multi‐disciplinary collaboration. In much the same way, collaboration between stakeholders, managers, decision‐makers and ecologists enables efficient uptake and implementation of management recommendations.

## Author Contributions

Cassie N. Speakman lead the manuscript. Cassie N. Speakman, Olivier Gimenez, Sarah Bull, Sarah Cubaynes, Katrina J. Davis, Sébastien Devillard, John M. Fryxell, Cara A. Gallagher, Elizabeth A. McHuron, Kévan Rastello, Isabel M. Smallegange and Roberto Salguero‐Gómez wrote the first draft of the manuscript. All authors contributed substantially to revisions.

## Peer Review

The peer review history for this article is available at https://www.webofscience.com/api/gateway/wos/peer‐review/10.1111/ele.70198.

## Supporting information


**Data S1:** ele70198‐sup‐0001‐supinfo.docx.

**Data S2:** ele70198‐sup‐0002‐supinfo.docx.

## Data Availability

No data or code were used in this manuscript.
