# Supplementary material for: Understanding and Predicting Population Response to Anthropogenic Disturbance: Current Approaches and Novel Opportunities
Source: Ecol Lett. 2025 Aug 22;28(8):e70198. doi: 10.1111/ele.70198 (PMC12374093; doi:10.1111/ele.70198)
Supplement: Supplementary file 1 — Data S1: ele70198‐sup‐0001‐supinfo.docx. [file ELE-28-0-s001.docx]

## Electronic Supplementary Material 1

Understanding and predicting population response to anthropogenic disturbance: Current approaches and novel opportunities

Cassie N Speakman^1,3,^*, Sarah Bull^2^, Sarah Cubaynes^3^, Katrina Davis^2^, Sébastien Devillard^4^, John Fryxell^5^, Cara A Gallagher^6^, Elizabeth A McHuron^7^, Kévan Rastello^8^, Isabel Smallegange^9^, Rob Salguero-Gomez^2^, Elsa Bonnaud^10^, Christophe Duchamp^11^, Patrick Giraudoux^12^, Simon Lacombe^3^, Courtney Marneweck^13^, Louis Schroll^3^, Adrien Tableau^11^, Sandrine Ruette^11,†^, Olivier Gimenez^3,†^

**Where to get started with modelling human impacts on animal populations**

Where available, we provide reference to useful resources for the different modelling approaches. In some cases, we may not be aware of resources for specific approaches. These are organised in the same manner as in the main text for ease of reference.

**Energetics models**

In-depth discussions on integration of energetics with ecological models, see Klansjscek *et al.* (2008), Nisbet *et al.* (2012), and van der Meer (2006).

*Traditional bioenergetic models*

Traditional bioenergetic models encompass a wide range of approaches. Some starting points include van der Meer (2006) and Sibly *et al.* (2013).

### Dynamic energy budget models

Dynamic energy budget (DEB) theory is very well documented with many resources including (Kooijman 2010) and approach specific references (within the main manuscript). Additionally, the Add-my-Pet portal (<https://www.bio.vu.nl/thb/deb/deblab/add_my_pet/>) contains data on the energetics of animal species required to parameterise DEB models, as well as a range of other resources.

## Individually-focused dynamics

### Individual-based models

There are many resources for getting started with individual-based models (IBMs or agent-based models, ABMs) including general reviews and considerations (Radchuk *et al.* 2016, 2021; Railsback & Grimm 2019) and recommended reporting practices (Grimm *et al.* 2020; Szangolies *et al.* 2024). There are several software platforms that are commonly used including NetLogo (<https://ccl.northwestern.edu/netlogo/>), RangeShifter (<https://rangeshifter.github.io/>), and HexSim (<https://www.hexsim.net/>), among others. Each of these software platforms have tutorials and guides.

Most of the information on how to use stochastic dynamic programming in ecology is provided in (Houston *et al.* 1988; Mangel & Clark 1988) and subsequent material from these authors. Use of stochastic dynamic programming for management decisions are well explained in (Marescot *et al.* 2013).

### Cell lattice models

For general information on cell-lattice models, see Bascompte & Solé (1995).

## Population dynamics

### Structured population models

Due to their relative simplicity, there are many resources available for matrix population models including extensive textbooks (Caswell 2001), book chapters (Koons *et al.* 2021; Vindenes *et al.* 2021) and methodology papers (Barraquand & Gimenez 2019; Shyu & Caswell 2018). There is also an extensive databases that can be used to parameterise matrix models for animals (Salguero-Gómez *et al.* 2016) and plants (Salguero‐Gómez *et al.* 2015).

The key resources for getting started with integral population models include (Merow *et al.* 2014; Rees *et al.* 2014). Software packages are available including R package *IPMpack* (Metcalf *et al.* 2013), while the *Rpadrino* R package (Levin *et al.* 2022) can be used to download data for integral projection models. An entry-level guide to using dynamic energy budget integral projection models (DEB-IPMs), as well as a dataset to parametrise DEB-IPMs for >180 ectotherm species, exists (Smallegange & Lucas 2024).

### Hierarchical models

Several comprehensive reviews of state-space models in ecological time series modeling are available, which comprise in-depth tutorial about model fitting and validation with examples in R (Auger-Méthé *et al.* 2021; Buckland *et al.* 2004). State-space models are commonly implemented using Bayesian methods often coded in R with packages like Rjags (Finley 2013) and nimble (de Valpine *et al.* 2017). Alternatively, maximum likelihood methods can also be used (Karban & De Valpine 2010).

A comprehensive introduction to integrated population models can be found in (Schaub & Kéry 2021), which covers the theory, examples, code, and data for self-study.

### Continuous time models

A comprehensive introduction to partial differential equations for ecologists can be found in Holmes *et al.* (1994). Also see Otto and Day (2011) and Moorcroft and Lewis (2006).

There are limited resources available on physiologically structured population models (Metz & Diekmann 1986; de Roos 1997). A software package for the numerical analysis of physiologically structured population models (PSPManalysis) is detailed in de Roos (2021).

Metapopulation models

Essential readings include Hanski (1999), which offers an in-depth exploration of metapopulation theory and models.

## Range dynamics

### Species distribution models

There are many resources available to get started with correlative species distribution models (SDM), including guidelines on the application of SDMs (Guisan *et al.* 2017; Sillero *et al.* 2021), common pitfalls (Araújo & Peterson 2012; Soley-Guardia *et al.* 2024), and reporting recommendations (Zurell *et al.* 2020).

To begin with machine learning models, ecologists can refer to Olden *et al.* (2008) for an introduction and applications, while Zhu *et al.* (2023) provides guidance on common pitfalls and best practices, and Desprez *et al.* (2023) offers insights on implementation and classification challenges. Extensive libraries and software packages for ML are available across multiple programming languages. A curated and maintained list can be found in the repository 'Awesome Machine Learning' (<https://github.com/josephmisiti/awesome-machine-learning>). Also see (Pichler & Hartig 2023) for a recent review on machine learning models.

### Process-explicit range models

Process-explicit range models (PEM) approaches, including eco-physiological models (Kearney & Porter 2009), coupled SDM-population models (Keith *et al.* 2008) and occupancy dynamics models (Kéry *et al.* 2013), have recently been reviewed in Briscoe *et al.* 2019), including recommendations on where to start with these approaches.

## Community dynamics

### Metacommunity models

Leibold *et al.* (2004) provides an overview of metacommunity approaches.

### Food web models

An introduction to food web models can be found in McCann (2011). Further resources are available for general food web models (*e.g.*, Pimm *et al.* 1991; Rossberg 2013) and Ecopath with Ecosim models (*e.g.*, Colléter *et al.* 2015; Heymans *et al.* 2016).

**References**

Araújo, M.B. & Peterson, A.T. (2012). Uses and misuses of bioclimatic envelope modeling. *Ecology*, 93, 1527–1539.

Auger-Méthé, M., Newman, K., Cole, D., Empacher, F., Gryba, R., King, A.A., *et al.* (2021). A guide to state–space modeling of ecological time series. *Ecological Monographs*, 91, e01470.

Barraquand, F. & Gimenez, O. (2019). Integrating multiple data sources to fit matrix population models for interacting species. *Ecological Modelling*, 411, 108713.

Bascompte, J. & Solé, R.V. (1995). Rethinking complexity: modelling spatiotemporal dynamics in ecology. *Trends in Ecology & Evolution*, 10, 361–366.

Briscoe, N.J., Elith, J., Salguero-Gómez, R., Lahoz-Monfort, J.J., Camac, J.S., Giljohann, K.M., *et al.* (2019). Forecasting species range dynamics with process-explicit models: matching methods to applications. *Ecology Letters*, 22, 1940–1956.

Buckland, S.T., Newman, K.B., Thomas, L. & Koesters, N.B. (2004). State-space models for the dynamics of wild animal populations. *Ecological Modelling*, 171, 157–175.

Caswell, H. (2001). *Matrix population models*. Sinauer Associates.

Colléter, M., Valls, A., Guitton, J., Gascuel, D., Pauly, D. & Christensen, V. (2015). Global overview of the applications of the Ecopath with Ecosim modeling approach using the EcoBase models repository. *Ecological Modelling*, 302, 42–53.

Desprez, M., Miele, V. & Gimenez, O. (2023). Nine tips for ecologists using machine learning.

Finley, A.O. (2013). Using JAGS in R with the rjags package.

Grimm, V., Railsback, S.F., Vincenot, C.E., Berger, U., Gallagher, C., DeAngelis, D.L., *et al.* (2020). The ODD Protocol for Describing Agent-Based and Other Simulation Models: A Second Update to Improve Clarity, Replication, and Structural Realism. *JASSS*, 23, 7.

Guisan, A., Thuiller, W. & Zimmermann, N.E. (2017). *Habitat suitability and distribution models: with applications in R*. Cambridge University Press.

Hanski, I. (1999). *Metapopulation ecology*. Oxford University Press.

Heymans, J.J., Coll, M., Link, J.S., Mackinson, S., Steenbeek, J., Walters, C., *et al.* (2016). Best practice in Ecopath with Ecosim food-web models for ecosystem-based management. *Ecological modelling*, 331, 173–184.

Holmes, E.E., Lewis, M.A., Banks, J. & Veit, R. (1994). Partial differential equations in ecology: spatial interactions and population dynamics. *Ecology*, 75, 17–29.

Houston, A., Clark, C., McNamara, J. & Mangel, M. (1988). Dynamic models in behavioural and evolutionary ecology. *Nature*, 332, 29–34.

Karban, R. & De Valpine, P. (2010). Population dynamics of an Arctiid caterpillar–tachinid parasitoid system using state-space models. *Journal of Animal Ecology*, 79, 650–661.

Kearney, M.R. & Porter, W. (2009). Mechanistic niche modelling: combining physiological and spatial data to predict species’ ranges. *Ecology Letters*, 12, 334–350.

Keith, D.A., Akçakaya, H.R., Thuiller, W., Midgley, G.F., Pearson, R.G., Phillips, S.J., *et al.* (2008). Predicting extinction risks under climate change: coupling stochastic population models with dynamic bioclimatic habitat models. *Biology Letters*, 4, 560–563.

Kéry, M., Guillera-Arroita, G. & Lahoz-Monfort, J.J. (2013). Analysing and mapping species range dynamics using occupancy models. *Journal of Biogeography*, 40, 1463–1474.

Kooijman, S.A.L.M. (2010). *Dynamic energy budget theory for metabolic organisation*. Cambridge University Press.

Koons, D.N., Iles, D.T. & Stott, I. (2021). Transient analyses of population dynamics using matrix projection models. In: *Demographic Methods across the Tree of Life* (eds. Salguero-Gomez, R. & Gamelon, M.). Oxford University Press, p. 0.

Leibold, M.A., Holyoak, M., Mouquet, N., Amarasekare, P., Chase, J.M., Hoopes, M.F., *et al.* (2004). The metacommunity concept: a framework for multi-scale community ecology. *Ecology Letters*, 7, 601–613.

Levin, S.C., Evers, S., Potter, T., Guerrero, M.P., Childs, D.Z., Compagnoni, A., *et al.* (2022). Rpadrino: An R package to access and use PADRINO, an open access database of Integral Projection Models. *Methods in Ecology and Evolution*, 13, 1923–1929.

Mangel, M. & Clark, C.W. (1988). *Dynamic Modeling in Behavioral Ecology*. Princeton University Press.

Marescot, L., Chapron, G., Chadès, I., Fackler, P.L., Duchamp, C., Marboutin, E., *et al.* (2013). Complex decisions made simple: a primer on stochastic dynamic programming. *Methods in Ecology and Evolution*, 4, 872–884.

McCann, K.S. (2011). *Food webs (MPB-50)*. Princeton University Press.

van der Meer, J. (2006). Metabolic theories in ecology. *Trends in Ecology & Evolution*, 21, 136–140.

Merow, C., Dahlgren, J.P., Metcalf, C.J.E., Childs, D.Z., Evans, M.E.K., Jongejans, E., *et al.* (2014). Advancing population ecology with integral projection models: a practical guide. *Methods in Ecology and Evolution*, 5, 99–110.

Metcalf, C.J.E., McMahon, S.M., Salguero‐Gómez, R. & Jongejans, E. (2013). IPM pack: an R package for integral projection models. *Methods in Ecology and Evolution*, 4, 195–200.

Metz, J.A.J. & Diekmann, O. (Eds.). (1986). *The Dynamics of Physiologically Structured Populations*. Lecture Notes in Biomathematics. Springer.

Moorcroft, P.R. & Lewis, M.A. (2006). *Mechanistic Home Range Analysis*. Princeton University Press.

Nisbet, R.M., Jusup, M., Klanjscek, T. & Pecquerie, L. (2012). Integrating dynamic energy budget (DEB) theory with traditional bioenergetic models. *Journal of Experimental Biology*, 215, 892–902.

Olden, J.D., Lawler, J.J. & Poff, N.L. (2008). Machine Learning Methods Without Tears: A Primer for Ecologists. *The Quarterly Review of Biology*, 83, 171–193.

Otto, S.P. & Day, T. (2011). *A biologist’s guide to mathematical modeling in ecology and evolution*. Princeton University Press.

Pichler, M. & Hartig, F. (2023). Machine learning and deep learning—A review for ecologists. *Methods in Ecology and Evolution*, 14, 994–1016.

Pimm, S.L., Lawton, J.H. & Cohen, J.E. (1991). Food web patterns and their consequences. *Nature*, 350, 669–674.

Radchuk, V., Kramer-Schadt, S., Berger, U., Scherer, C., Backmann, P. & Grimm, V. (2021). Individual-based models. In: *Demographic Methods across the Tree of Life* (eds. Salguero-Gomez, R. & Gamelon, M.). Oxford University Press, p. 0.

Radchuk, V., Oppel, S., Groeneveld, J., Grimm, V. & Schtickzelle, N. (2016). Simple or complex: Relative impact of data availability and model purpose on the choice of model types for population viability analyses. *Ecological Modelling*, 323, 87–95.

Railsback, S.F. & Grimm, V. (2019). *Agent-based and individual-based modeling: a practical introduction*. Princeton university press.

Rees, M., Childs, D.Z. & Ellner, S.P. (2014). Building integral projection models: a user’s guide. *Journal of Animal Ecology*, 83, 528–545.

de Roos, A.M. (1997). A gentle introduction to physiologically structured population models. In: *Structured-population models in marine, terrestrial, and freshwater systems*. Springer, pp. 119–204.

de Roos, A.M. (2021). PSPManalysis: Steady-state and bifurcation analysis of physiologically structured population models. *Methods in Ecology and Evolution*, 12, 383–390.

Rossberg, A.G. (2013). *Food webs and biodiversity: foundations, models, data*. John Wiley & Sons.

Salguero-Gómez, R., Jones, O.R., Archer, C.R., Bein, C., de Buhr, H., Farack, C., *et al.* (2016). COMADRE: a global data base of animal demography. *Journal of Animal Ecology*, 85, 371–384.

Salguero‐Gómez, R., Jones, O.R., Archer, C.R., Buckley, Y.M., Che‐Castaldo, J., Caswell, H., *et al.* (2015). The compadre Plant Matrix Database: an open online repository for plant demography. *Journal of Ecology*, 103, 202–218.

Schaub, M. & Kéry, M. (2021). *Integrated population models: Theory and ecological applications with R and JAGS*. Academic Press.

Shyu, E. & Caswell, H. (2018). Mating, births, and transitions: a flexible two-sex matrix model for evolutionary demography. *Population Ecology*, 60, 21–36.

Sibly, R.M., Grimm, V., Martin, B.T., Johnston, A.S.A., Kułakowska, K., Topping, C.J., *et al.* (2013). Representing the acquisition and use of energy by individuals in agent-based models of animal populations. *Methods in Ecology and Evolution*, 4, 151–161.

Sillero, N., Arenas-Castro, S., Enriquez‐Urzelai, U., Vale, C.G., Sousa-Guedes, D., Martínez-Freiría, F., *et al.* (2021). Want to model a species niche? A step-by-step guideline on correlative ecological niche modelling. *Ecological Modelling*, 456, 109671.

Smallegange, I.M. & Lucas, S. (2024). DEBBIES Dataset to study Life Histories across Ectotherms. *Sci Data*, 11, 153.

Soley-Guardia, M., Alvarado-Serrano, D.F. & Anderson, R.P. (2024). Top ten hazards to avoid when modeling species distributions: a didactic guide of assumptions, problems, and recommendations. *Ecography*, 2024, e06852.

Szangolies, L., Rohwäder, M.-S., Ahmed, H., Jahanmiri, F., Wagner, A., Souto-Veiga, R., *et al.* (2024). Visual ODD: A Standardised Visualisation Illustrating the Narrative of Agent-Based Models. *JASSS*, 27, 1.

de Valpine, P., Turek, D., Paciorek, C.J., Anderson-Bergman, C., Lang, D.T. & Bodik, R. (2017). Programming with models: writing statistical algorithms for general model structures with NIMBLE. *Journal of Computational and Graphical Statistics*, 26, 403–413.

Vindenes, Y., Le Coeur, C. & Caswell, H. (2021). Introduction to matrix population models. In: *Demographic Methods across the Tree of Life* (eds. Salguero-Gomez, R. & Gamelon, M.). Oxford University Press, p. 0.

Zhu, J.-J., Yang, M. & Ren, Z.J. (2023). Machine Learning in Environmental Research: Common Pitfalls and Best Practices. *Environ. Sci. Technol.*, 57, 17671–17689.

Zurell, D., Franklin, J., König, C., Bouchet, P.J., Dormann, C.F., Elith, J., *et al.* (2020). A standard protocol for reporting species distribution models. *Ecography*, 43, 1261–1277.
