# Supplementary material for: Understanding and Predicting Population Response to Anthropogenic Disturbance: Current Approaches and Novel Opportunities
Source: Ecol Lett. 2025 Aug 22;28(8):e70198. doi: 10.1111/ele.70198 (PMC12374093; doi:10.1111/ele.70198)
Supplement: Supplementary file 2 — Data S2: ele70198‐sup‐0002‐supinfo.docx. [file ELE-28-0-s002.docx]

## Electronic Supplementary Material 2

Understanding and predicting population response to anthropogenic disturbance: Current approaches and novel opportunities

Cassie N Speakman^1,3,^*, Sarah Bull^2^, Sarah Cubaynes^3^, Katrina Davis^2^, Sébastien Devillard^4^, John Fryxell^5^, Cara A Gallagher^6^, Elizabeth A McHuron^7^, Kévan Rastello^8^, Isabel Smallegange^9^, Rob Salguero-Gomez^2^, Elsa Bonnaud^10^, Christophe Duchamp^11^, Patrick Giraudoux^12^, Simon Lacombe^3^, Courtney Marneweck^13^, Louis Schroll^3^, Adrien Tableau^11^, Sandrine Ruette^11,†^, Olivier Gimenez^3,†^

**Model inputs and outputs**

**ESM Table 1.** Data inputs and outputs of different modelling approaches for investigating the impacts of human disturbance on animal populations.

| **Modelling approach** | **Inputs** | **Outputs** |
| --- | --- | --- |
| **Individually-focused** | | |
| Individual-based models | Application specific | Population dynamics based on individual interactions |
|  | Individual-level parameters where possible | Can produce spatial patterns of individuals and groups across time |
|  | For estimating population level responses to stressors, at least need vital rates estimates |  |
| Stochastic dynamic programming | Fitness function linking state variables to fitness | Optimal strategies for survival or reproduction under uncertain conditions |
|  | Often information about the prey landscape | Expected population trajectories under disturbance scenarios |
| Cell-lattice models | Spatially explicit grid data (species occurrence or abundance) | Spatially explicit population dynamics over time |
|  | Data on disturbance impacts (*e.g.*, intensity, spatial extent) | Effects of local disturbances on population persistence or spread |
| **Population dynamics** |  |  |
| Matrix population models | Life stage-specific survival and fecundity/reproductive rates | Population size and population growth rate |
|  | Transition matrices (*e.g.*, for survival, reproduction) by stage class | Stable (st)age distribution |
| Integral projection models | Continuous data on individual traits (*e.g.*, size, age, condition) | Population growth rates based on continuous individual trait distributions |
|  | Survival, growth, and reproduction functions for trait changes | Stage or size structure across generations |
| State-space models | Time-series data on population abundance or demographic parameters | Predictions of population trends over time |
|  |  | Estimates of hidden (latent) population states (*e.g.*, true population size) |
| Integrated population models | Count data on abundance | Integrated estimates of population size, survival, and reproduction |
|  | Individual-based data (preferably age-specific) on survival and/or fecundity |  |
| Partial differential equations | Spatial and temporal data on population densities or distributions | Continuous spatial and temporal population dynamics |
|  | Environmental factors affecting population growth or diffusion | Predictions of population spread or contraction under disturbance regimes |
| Physiologically structured population models | State variables for individuals (*e.g.*, age, energy, size) | Predictions of population structure under environmental stress |
|  | State-dependent survival and reproduction rates | Population dynamics driven by physiological constraints |
|  | Developmental rules (ODEs describing growth, maturation) | Evolutionarily stable strategies, population structure, trait dynamics |
|  | Feedback from population to environment (*e.g.*, resource depletion) |  |
| Metapopulation models | Data on local population sizes, habitat connectivity, and patch dynamics | Persistence of metapopulations across multiple patches |
|  | Dispersal rates between habitat patches | Effects of disturbance on patch occupancy and metapopulation viability |
| **Range dynamics** | | |
| Species distribution models | Occurrence or abundance data | Predicted species distributions across landscapes |
|  | Spatially-explicit climatic and landscape data |  |
| Process explicit range models | Data requirements vary between PERMs | Outputs vary between PERMs; see Briscoe *et al.* (2019) for details |
|  | Spatially-explicit climatic and landscape data |  |
| **Community and ecosystem dynamics** | | |
| Metacommunity models | Species interactions (*e.g.*, competition, predation) | Dynamics of multiple interacting communities across a landscape |
|  | Habitat connectivity and environmental gradients | Effects of disturbance on community composition and structure |
| Food web models | Species interactions (*e.g.*, predation, competition, mutualism) | Stability and resilience of the food web |
|  | Biomass or abundance of species within the food web | Impacts of disturbances on trophic structure and species interactions |
|  | Flow of energy and nutrients through the system |  |
